# Organoleptic properties and neuroimaging response on the perception of edible gels

**DOI:** 10.1016/j.heliyon.2025.e41649

**Published:** 2025-01-02

**Authors:** Jakub Berčík, Vladimír Vietoris, Melina Korčok, Adriana Rusková, Ján Durec, Katarína Neomániová

**Affiliations:** a Institute of Marketing, Trade and Social Studies, Faculty of Economics and Management, Slovak University of Agriculture, 949 76, Nitra, Slovakia; b Institute of Food Sciences, Faculty of Biotechnology and Food Sciences, Slovak University of Agriculture, 949 76, Nitra, Slovakia; c McCarter, Inc., 821 01, Bratislava, Slovakia

**Keywords:** Foodgels, Electroencephalography, Facereading, Sensory affective testing, Temporal dominance of sensations (TDS) technique

## Abstract

The rapidly increasing number of elderly people in the world highlights the need for the development of innovative foods with modified textures that do not expose the elderly to the risks associated with food consumption (risk of aspiration, suffocation, and chocking). Providing specific food such as edible gel for the elderly population and the study of their properties is a challenge for the scientific community. There are some available gels in the supermarkets destined for the sports population, with specific texture and technological properties that could be used and extrapolated for senior people. To explore this potential, five types of sport commercial gels purchased from a local Slovak market were characterized in order to evaluate their technological properties and to know if these types of gels are suitable for the senior population. The energy gels were evaluated using acceptance testing, involving 75 seniors who evaluated important organoleptic attributes by a combination of hedonic and intensity scales. The same consumer panel then profiled the gels using the Temporal Dominance of Sensations (TDS) technique. The prevalence of food neophobia was measured with the Food Neophobia Scale (FNS) and also using neuroimaging and biometric methods. The results suggest that there are significant differences in the perception of edible gels, as confirmed by measurements via electroencephalography (EEG) and Facereading. We conclude by suggesting the potential of specific foods such as edible gels for the elderly population as our findings also confirm that the composition of these specific and sustainable foods may elicit different perceptions. This highlights the need to use biometric and neuroimaging methods in food research in order to create more optimal formulations for specific populations.

## 1 Introduction

Population ageing has become an important global concern. The elderly aged 65 years and over will represent 12 % (by 2030) and 16 % (by 2050) of the total world population compared to 10 % in 2022 [1]. Older age is associated with problems affecting the digestive, respiratory, nervous, cardiovascular, and excretory systems, as well as metabolic problems. Physical activity decreases with age, leading to muscle wasting and requiring dietary modification [[2], [3], [4], [5]]. Ensuring healthy aging is an important challenge for our society [6]. Addressing the problems associated with malnutrition in the elderly is essential to reduce mortality and prolong survival, as the prevalence of malnutrition in the elderly is high [[7], [8], [9]]. Taste loss, decreased olfaction, dry mouth, presbyphagia, hyposalivation, teeth loss, and distribution of remaining teeth all contribute to altered food intake. These anatomical and physiological changes associated with older age affect the processes of chewing, swallowing, and feeding, the satisfaction of an individual's nutritional needs, as well as the choice and quality of consumed foods [[10], [11], [12], [13]].

Therefore, it is essential to develop innovative and functional foods [2,14]. Consideration of the structure of the food bolus when developing easy-to-eat and easy-to-swallow foods for seniors emerges as important [13]. Foods for the elderly should be soft, safe, and easy to swallow [11]. Relatively new products, such as lubricating gels or liquids designed to make oral medications easier to swallow, are appearing on the market, especially for people who have swallowing difficulties [15]. Special foods for the elderly include thickened liquids and foods with modified texture. Gels or purees are soft foods that disintegrate easily in the mouth [16]. As soft gels do not require chewing when consumed, they represent a suitable form of food for the elderly and can be safely ingested. Consumption consists of squeezing between the tongue and the hard palate without mastication [[17], [18], [19], [20], [21]]. For example, a pea protein gel containing 41.7 % of the protein in dry matter, of which the texture is transformed from thick to soft solid by microwave heating is suitable for patients with dysphagia [22], or the elderly [16]. Hydrogels are well suited for the development of foods tailored to the needs of the elderly, with some polysaccharides, proteins, and lipids already being used in creating texture-modified foods designed for the elderly with chewing and swallowing challenges (thick liquids, jellies, creams, creams, sauces, foams, among others). In the food industry, the use of natural polymer hydrogels is also well established in the development of food for the elderly and 3D food printing applications [[23], [24], [25], [26]]. Certain foods with modified textures and attractive sensory properties, such as edible gels, could provide an alternative to conventional foods to ensure daily intake of macro and micronutrients to elderly populations [23,27].

Foods for the elderly should have pleasing sensory properties in order to achieve consumer acceptability. Hardness, cohesiveness, and adhesion are important textural properties for safe swallowing [28,29]. Different approaches have been chosen for the evaluation of the sensory properties of gels or gel-based/gel-infused foods foods e.g., descriptive analysis of 3D-printed yogurt gels using a trained panel [30], Check-All-That-Apply (CATA) of milkshakes with added hydrocolloids using untrained consumers [31], descriptive analysis of a mango gel snacks using a trained panel [32], acceptance testing of artificial steak using consumers and a seven-point hedonic scale [33,34] quantitative descriptive analysis (QDA) of food gels prepared from aqueous extract of fish scales using a trained panel [34], QDA was used for the evaluation of eating difficulties using hydrocolloids as a model food product and a trained panel [35]. Igarashi et al. [36] focus on rheological and textural properties (viscosity and hardness) to evaluate swallowing amplitude on food to decrease dysphagic in some patients since varying viscosity of food, had been associated with changes in swallowing parameters in these populations.

In order to make progress in this area, studies of available gel foods on the market should be carried out to investigate the characteristics, as well as sensory preferences of the elderly population towards gel foods. The aim of this work was to investigate the selected gel products available on the market with an emphasis on sensory aspects, which can be further used in the development of edible gel products specially modified for seniors.

## 2 Materials and methods

### 2.1 Ethical considerations and participants

**Fig. 1:**
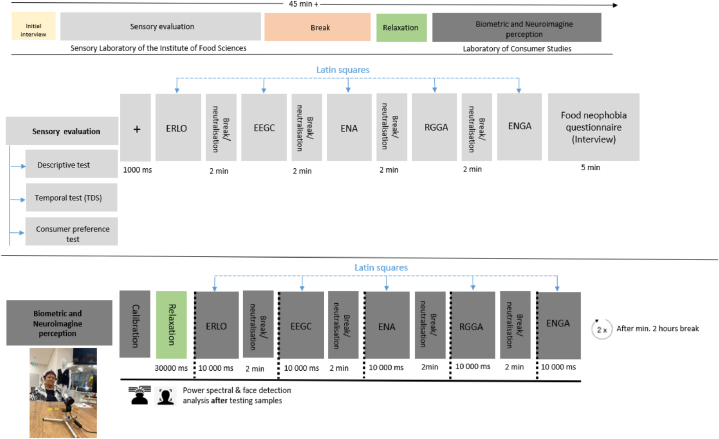
The time course of the testing. Note: TDS – temporal dominance of senses, ERLO – commercial energy gel with lemon-orange flavor; EEGC – commercial energy gel with cherry flavor; ENA – commercial energy gel with apricot flavor; RGGA – commercial energy gel with green apple flavor; ENGA – commercial energy gel with green apple flavor.

A total of 84 participants were recruited from the Institute's pool of participants. Prior to the realization of the study, ethical approval was granted by the Ethical Committee of the Slovak University of Agriculture in Nitra (SPU), registered under the number 2022-016. All participants provided informed consent in accordance with the Code of Ethics of the "Laboratory of Consumer Studies" at the Faculty of Economics and Management of the Slovak University of Agriculture in Nitra [37]. 84 recruited participants met the age quotas, but due to technical problems and incorrect recording of neuroimaging and biometric data, 9 respondents were excluded. Therefore, only 75 participants (36 female) were in further analyses. For studies that combine sensory assessment with biometric and neuroimaging tools, this is a sufficient number of respondents [38,39]. The participants were aged between 50 and 70 years (M = 55.6, SD = 5.4) with a Body Mass Index (BMI) ranging from 18.4 to 56.4 (M = 32.6, SD = 4.2). On average, 1.75 h (SD = 1.9) had passed since their last meal. Respondents took part in the evaluation in two separate laboratories, see Fig. 1, and repeated the procedure in the Laboratory of Consumer Studies over time. Only one respondent was in the laboratory with the researcher at any given time during the sample evaluation. Before the evaluation, each participant signed a consent form informing them of the potential risks of the evaluation. The evaluation was voluntary and participants could leave the study at any stage without giving a reason.

### 2.2 Samples

Based on market research, 5 samples of energy gels for athletes were selected that are designed to provide quick energy before or after training. The samples were packaged in 75g or 80g packs. The detailed composition of the commercial gels evaluated as well as the flavors are presented in Table 1.

**Table 1:** The composition and flavors of evaluated samples.

Sample	Composition	Energy value/100g	Flavor
ERLO	Water, citric acid, flavor, xanthan and guar gum, sucralose, potassium sorbate and sodium benzoate, maltodextrine, dextrose, Palatinose™ (isomaltulose), L-glutamine, taurine, L-leucine, trimagnesium citrate, calcium lactate, potassium citrate, L-isoleucine, L-valine, L-carnitine base, L-alanine, beta alanine, L-citrulline DL malate, arginine AKG, creatine ethyl ester, tri-creatine malate, monopotassium phosphate, sodium chloride, inosin, vitamin premix (vitamin C, B1, B2, B3, B5, B6, folic acid, biotin, vitamin B12), sodium citrate.	607 kJ/145 kcal	Lemon-orange
ENGA	Water, Palatinose™ (isomaltulose), glucose, xanthan gum, taurine, glycine, L-leucine, L-alanine, citric acid and malic acid, L-isoleucine, L-valine, flavoring, sodium benzoate and sorbic acid, L-carnosine, acesulfame K and sucralose.	666 kJ/157 kcal	Green apple
EEGC	Water, xanthan, citric acid, flavour, sucralose, potassium sorbate and sodium benzoate, dextrose, maltodextrine, Palatinose™ (isomaltulose), taurine, potassium citrate, calcium lactate, L-tyrosine, trimagnesium citrate, sodium citrate, sodium chloride, caffeine anhydrous.	778 kJ/185 kcal	Cherry
RGGA	Water, Palatinose™ (isomaltulose), glucose, xanthan gum, taurine, glycine, L-leucine, L-alanine, citric acid, malic acid L-isoles, flavorings, sodium benzoate, sorbic acid, L-carnosine, acesulfame K, sucralose.	500 kJ/118 kcal	Green apple
ENA	Water, Palatinose™ (isomaltulose), glucose, xanthan gum, taurine, glycine, L-leucine, L-alanine, citric acid and malic acid, L-isoleucine, L-valine, flavoring, sodium benzoate and sorbic acid, L-carnosine, acesulfame K and sucralose.	666 kJ/157 kcal	Apricot

The gels were stored in dry conditions, with storage temperatures not exceeding 25 °C (as recommended by the manufacturers on the product packaging). They were served to the evaluators without any treatment.

### 2.3 Sensory evaluation

**Fig. 2:**
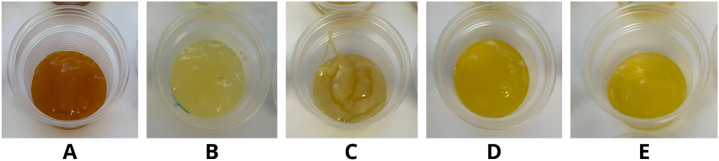
Visual depiction of served samples: A = ERLO – commercial energy gel with lemon-orange flavor; B = ENGA – commercial energy gel with green apple flavor; C = EEGC – commercial energy gel with cherry flavor; D = RGGA – commercial energy gel with green apple flavor; E = ENA – commercial energy gel with apricot flavor.

The evaluation of 5 commercial gels was conducted in the sensory laboratory of the Institute of Food Sciences (Slovak University of Agriculture in Nitra, Nitra, Slovakia) developed in accordance with the requirements of the ISO 8589:2007 guidelines. A plastic cup received an administration of 10–15 ml of the sample, and each sample was labeled with a three-digit code. The samples were presented following the randomized complete block design (for all analyses). Fig. 2 illustrates the administered samples, comprising the following: ERLO – a commercial energy gel with lemon-orange flavor (Fig. 2A); ENGA – a commercial energy gel with green apple flavor (Fig. 2B); EEGC – a commercial energy gel with cherry flavor (Fig. 2C); RGGA – a commercial energy gel with green apple flavor (Fig. 2D); and ENA – a commercial energy gel with apricot flavor (Fig. 2E).

The descriptors were selected based on the sensory evaluation of similar products [30,31,33,34,40]. For the evaluation of the overall liking in the sections appearance, taste, and texture during consumption as well as for the evaluation of the overall acceptance, the 9-point hedonic scale from 1-“dislike extremely” to 9-“like extremely” was used [41]. A 9-point intensity scale ranging from 1-''none'' to 9-''very high'' was used for the evaluation of other attributes.

Due to the broadness of the evaluated attributes, the consumer test questionnaire was divided into 10 parts (appearance, color, smell, texture before consumption, sensation in the mouth, taste, flavor, texture during consumption, aftertaste, and overall acceptance) and their definitions, evaluation techniques are described in Table 2, Table 3. Due to the complexity of the evaluation of the textural attributes before consumption, they have been described in a separate table (Table 3). Only for attributes related to appearance, including fluidity, were these archer points explained for attribute fluidity from 1-“very liquid to 9-“creamy”; homogeneity from 1-“particle separated” to 9-“homogeneous mixture” and opalescence from 1-“matt” to 9-“radiant” acceptance. In addition, claims such as it is easy to swallow, it is a healthy snack, it could replace food, I would eat it as a late dinner, I would eat it as an afternoon snack, and I would eat it for breakfast, were questioned. Evaluators expressed their agreement with the following statements on a 9-point Likert scale ranging from 1-“completely disagree”; 3-”disagree”; 5-“neutral”; 7-“agree”, 9-“completely agree”.

**Table 2:** Definition of evaluated attributes and evaluation techniques.

Section	Definition	Evaluation technique	Attributes	Scale
Appearance	A sense impression of external aspects of samples.	Assessment or observation of a sample before consumption.	Fluidity	1 “very liquid to 9 “creamy”;
			Homogeneity of the gel	1 “particle separated” to 9-“homogeneous mixture”
			Opalescence	1 “matt” to 9-“radiant”
			Overall liking	1-“dislike extremely” to 9-“like extremely”
Color	The appearance of samples in terms of hue, lightness, and saturation.	Assessment or observation of a sample before consumption.	Artificial Natural	1-''none'' to 9-''very high''
Smell	Odors or fragrances which the sample leaves behind/exhibits on flavoring.	Sniffing the sample before consumption.	Artificial Natural Fruity smell	1-''none'' to 9-''very high''
Sensation in the mouth	Physical sensations that are evoked by samples in the mouth.	Tasting and sensation perception in the mouth.	Smooth texture Melting of the gel in the mouth	1-''none'' to 9-''very high''
Taste	The taste sensation that arises on contact with the sample perceived in the mouth and throat.	By taking part of the sample with a spoon, putting it in the mouth, holding it for a few seconds and then swallowing it.	Sweet Bitter Sour Artificial	1-''none'' to 9-''very high''
			Overall liking	1-“dislike extremely” to 9-“like extremely”
Flavor	The complex taste sensation of flavors and aromas.	Tasting the gel.	Fruity Artificial	1-''none'' to 9-''very high''
Texture during consumption	Sensory manifestation of the structure of the sample or its inner composition.	Assessment of the sample texture and mouthfeel upon consumption.	Jelly like Adhesives Fine Granular Oily Liquid	1-''none'' to 9-''very high''
			Overallliking	1-“dislike extremely” to 9-“like extremely”
Aftertaste	The taste after swallowing the sample.	Assessment of retained taste after ingestion of the sample.	Astringent Bitter Artificial	1-''none'' to 9-''very high''
Overall acceptance	Overall likeability and acceptance of evaluated samples.	By considering all the evaluated attributes, the likeability of samples, but also the individual preference of the consumer.	Overall liking and acceptability of the product	1-“dislike extremely” to 9-“like extremely”

**Table 3:** Definition of evaluation techniques for attributes included in the section texture before consumption.

Attribute	Definition	Evaluation technique	Scale
Springiness	Degree of return of the sample to its original state after squeezing with a spoon.	Moving the sample with a spoon and visually assessing its degree of return.	1-''none'' to 9-''very high''
Firmness	The force required to break/split the sample in the plastic container with a spoon.	Crushing the gel layer with a spoon and evaluating the necessary strength.	1-''none'' to 9-''very high''
Adhesion to the spoon	Degree of movement of the sample in a teaspoon inclined at 45°.	Scooping the sample onto the teaspoon, rotating the spoon by 45 °C and visually observing the amount of dropped sample.	1-''none'' to 9-''very high''
Surface smoothness	Visual perception of the shape and size of particles on the gel surface.	Assessment or observation of a sample before consumption.	1-''none'' to 9-''very high''

### 2.4 Temporal dominance of sensations (TDS)

Seventy-five evaluators participated in the evaluation of edible gels using the TDS method. Participants were instructed to press the start button immediately after inserting the gel into their mouths and then to consider which of the attributes offered by the software, they perceived to be dominant and to rate their intensity. If this changed, participants were instructed to record the next dominant attribute [42]. The duration of the evaluation was 15 s. Subsequently, raters were asked to use water and pieces of white bread for palate cleansing between samples. Data capturing was carried out using Sensomaker (version 1.8) software [43].

### 2.5 Viscosity measurement and texture analysis

Brookfield viscosimeter Model DV2T HA Brookfield Engineering Laboratories, Inc, Middleboro, MA, USA was used for the measurement of dynamic viscosity. The viscosimeter was equipped with spindle T-B (92), and the measurements were carried out at a temperature of 20 °C and a speed of 200 min⁻^1^. Based on the magnitude of the deflection, the instrument recalculates the dynamic or apparent viscosity. The measurements were carried out in three repetitions.

Texture analysis of commercial gels was performed using a compression test with a TA.XT.plus texture analyzer (Stable Micro Systems, Surrey, UK). The texture analyzer was equipped with a 5 kg load cell and a cylindrical stainless-steel back-extrusion probe with a diameter of 35 mm was introduced in 40 g of the sample and placed into a standard back-extrusion container (40 mm diameter) at a speed of 1 mm/s. Two repetitions of the measurements were performed. The following attributes were investigated: firmness, consistency, cohesiveness, and work of cohesion while the samples were tempered to laboratory temperature (22 °C ± 1 °C).

### 2.6 Food neophobia, measuring emotions by neuroimaging (brain measurement), and biometric (face recognition)

**Fig. 3:**
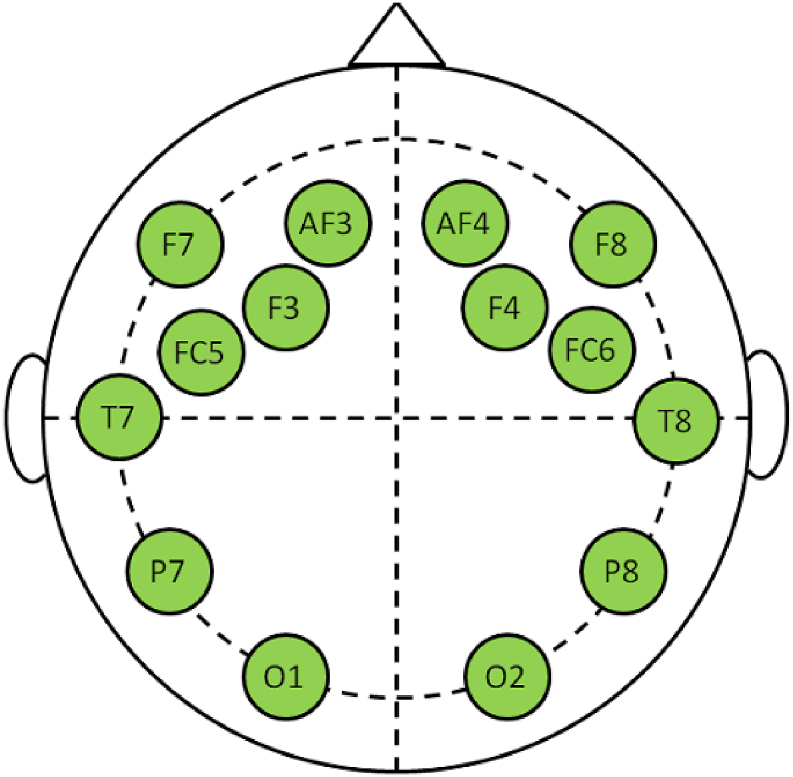
Emotiv EPOC headset — 10–20 system.

The Food Neophobia Questionnaire scored on a 7-point scale (1 for stronger agreement and 7 for stronger disagreement), included ten statements assessing willingness to try novel foods, with the total score ranging from 10 to 70 [44]. A score of 38.6 or higher indicates a high level of food neophobia, which is characterized by a reluctance to try new foods, while a score of 16.4 or lower indicates a strong willingness and enthusiasm to try new foods [38]. The EEG headset from company Emotiv EPOC+, equipped with 14 data and 2 reference electrodes, measured brain activity using the international 10–20 electrode distribution system. The electrodes were positioned at AF3, F7, F3, FC5, T7, P7, O1, O2, P8, T8, FC6, F4, F8, and AF4, as depicted in Fig. 3, adhering to international standards [45,46]. The reliability of data collected using the Emotiv EPOC mobile electroencephalograph (EEG) has been confirmed by multiple studies [[47], [48], [49], [50]], which demonstrated that it delivers results comparable to traditional stationary EEG devices. Calibration of the EEG equipment was also performed prior to testing in accordance with the scientific procedures of [51,52].

After calibration, the emotional response of the respondents was monitored as part of the sensory evaluation of the edible gels. The emotional response of interest was the emotional response in the 10s time range immediately after the end of the tasting session to avoid data bias (distracting artefacts arising from, e.g., chewing). The determination of the time interval was based on other studies [53] focusing on the attention span of participants.

The data recorded using EEG was processed using power spectrum analysis [54] in Emotiv PRO version 1.356 [55].

Emotional engagement and excitement, two fundamental emotional states, were observed. Electrical activity recorded by a set of electrodes required for the emotion calculation was used to calculate each person's emotion. EEG channels assigned to locations O1, O2, F3, F4, F7, F8, FC5, and FC6 are necessary for calculating emotional engagement. The variables for this model were profiled from related studies using gradual regression [[56], [57], [58]]. (BetaF3 + BetaF4)/(AlphaF3 + AlphaF4) was used to calculate excitement [59,60].

The data were collected using the Affective Suite software, which tracks real-time emotional changes. Each participant had a unique profile, with the data adjusted based on specific personality traits. These differences were summed and used to normalize the results.

Emotions play a significant role in food perception and behavior [61] and various types of measures have been developed to assess food-induced emotions, most of which are based on subjective assessments (conscious feedback). It's important to recognize that individuals' personal frames shape how they perceive food stimuli, as they process sensory information, including unconscious influences. Therefore, assessing emotions related to food can be done through physiological measures or brain activity monitoring [62,63]. Emotional feedback (valence) of respondents was measured by observing changes in facial muscles and recognition of basic micro emotions including happiness, sadness, anger, disgust, surprise, fear, and neutrality through somatic biometric method Facereader 7 from the Dutch company Noldus (Wageningen, the Netherlands) [64]. Facial responses naturally reflect our emotional evaluations, even when exposed to emotional stimuli processed at the threshold of awareness [65]. These facial reactions, as well as changes in autonomic functions like sweat gland activity and heart rate, are indicative of valence-based responses, which are closely linked to arousal processes [66]. The valence ranges from pleasant to unpleasant and usually elicits behaviors in which the person approaches or withdraws, activating the appropriate motor responses [[67], [68], [69]].

The validity of the recorded data is largely dependent on the angle of scanning, ambient light, and the resolution of the recording device [70]. The data collected from individual measurements were synchronized and correlated with each other in the Noldus Observer XT 10 program environment. This program allows for the synchronize structured and unstructured data from individual devices while creating custom variables during the realization of experiments [64,71]. The research was conducted from 06 to June 10, 2022.

The Code of Ethics of the "Laboratory of Consumer Studies" at the Faculty of Economics and Management of the Slovak University of Agriculture in Nitra [37], as well as the ethical guidelines of the Neuromarketing Scientific and Business Association [72] and the ICC/ESOMAR Code of Ethics [73] were followed. Each participant was informed about the experimental procedure, received a short training on the methods used, and completed two forms following the GDPR: consent to biometric testing, and data processing and confirmation of training.

### 2.7 Statistical analysis

The descriptive analysis function in the RedJade Sensory Software (RedJade Sensory Solutions, LLC, Martinez, CA, USA) was used for the analysis of sensory data. The results were also evaluated in RStudio (version 2022.12.02 + 353, R Foundation for Statistical Computing, Vienna, Austria) using the SensoMiner package and the PCA method, and standard descriptive indices were also used to visualize the results. Statistical significance was calculated using One-Way Analysis of Variance (ANOVA) using XLSTAT software (New York, NY, USA, 2021). Sensomaker (version 1.8; [43]) software was used for data capturing and preparation of the TDS curves.

In the case of neuroimagine and biometric methods, all experiments were performed at least in two repetitions with a time interval and values were expressed as a mean ± standard deviation. All results were processed in the statistical program RStudio 2022. Statistical differences among different types of food gels were tested using ANOVA followed by LSD post-hoc analysis, in case of data gained from EEG and FaceReader. Data from EEG data were also tested, according to belonging to a group of respondents according to neophobic specification, using two samples T-test.

## 3 Results and discussion

### 3.1 Sensory evaluation

**Fig. 4:**
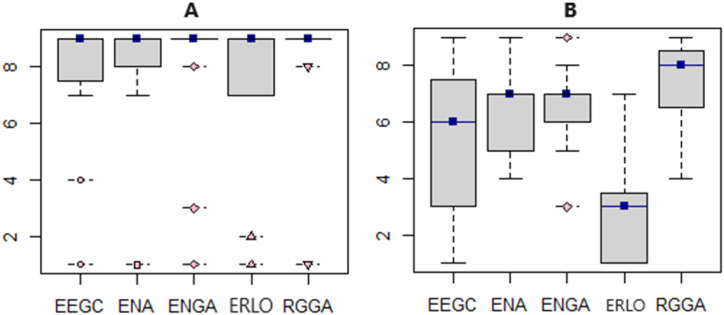
Sensory evaluation results for easy-to-swallow (A) and overall acceptance attribute (B).

The attributes characterizing the appearance of the gels (homogeneity, followed by opalescence (except EEGC sample)) were evaluated with a score exceeding 6 points. A statistically significant difference (p = 0.001) was observed for the homogeneity attribute. The most liked gel according to the evaluation of overall liking appearance was RGGA (7.40) gel followed by ENA (7.27), ENGA (6.87), ERLO (6.60), and EEGC (6.0). All commercial gels received high ratings for smooth texture, melting, and fine texture, with texture being one of the critical attributes in the preferences of consumers [74], the elderly [75], and dysphagia patients [76]. The results of the sensory evaluation are shown in Table 3. Overall, all of the energy gels were recognized by the evaluators as easy-to-swallow (see Fig. 4A). As the demand for the development of foods that are easy to swallow continues to grow with the increasing number of elderly people, some authors suggest that gels may be able to meet this demand [16,[77], [78], [79]]. In terms of overall acceptance (see Fig. 4B), the evaluators assigned the highest score to the RGGA sample (7.27), followed by ENGA (6.33) and ENA (6.27). Slightly lower scores were recorded for the EEGC sample (5.33) and the least preferred sample was ERLO (2.93).

ANOVA was performed (Table 4), where statistically significant differences were observed within the homogeneity, adhesion to the spoon, surface smoothness, artificial smell, natural smell, fruity smell, sour taste, sweet taste, artificial taste, overall liking of the taste, fruit flavor, artificial flavor, granular texture, artificial aftertaste, as well as overall acceptance attributes.

**Table 4:** Duncan and REGWQ tests (ANOVA) for sensory attributes.

	ENA	RGGA	ENGA	ERLO	EEGC	p-value	Scale
Fluidity	5.80 ± 1.94 a	6.00 ± 2.07 a	5.33 ± 1.78 a	6.93 a ±2.02 a	6.80 ± 2.07 a	0.171	1-“very liquid” to 9-“creamy”
**Homogeneity**	**8.40** ± **0.88 a**	**8.26** ± **1.24 a**	**7.6**7 ± **1.45 a**	**7.40** ± **1.62 a**	**5.87** ± **2.50 b**	**0.001**^a^	1-“particle separated” to 9-“homogeneous mixture”
Opalescence	7.20 ± 1.80 a	7.00 ± 1.63 a	6.47 ± 2.06 a	6.47 ± 1.96 a	6.93 ± 2.77 a	0.840	1-“matt” to 9-“radiant”
Overall liking appearance	7.27 ± 1.61a	7.40 ± 1.31 a	6.87 ± 1.41 a	6.60 ± 1.62 a	6.00 ± 1.75 a	0.131	1-“dislike extremely” to 9-“like extremely”
Artificial color	3.87 ± 2.22 a	4.13 ± 2.12 a	3.67 ± 2.18 a	4.93 ± 2.49 a	5.73 ± 2.77 a	0.133	1-''none'' to 9-''very high''
Natural color	5.80 ± 2.07 a	5.80 ± 2.01 a	6.60 ± 1.96 a	5.80 ± 2.59 a	4.33 ± 2.12 a	0.100	1-''none'' to 9-''very high''
Springiness	6.07 ± 2.05 a	5.93 ± 1.84 a	6.07 ± 1.69 a	5.27 ± 2.54 a	5.67 ± 2.67 a	0.853	1-''none'' to 9-''very high''
Firmness	3.60 ± 1.58 a	4.00 ± 2.03 a	3.93 ± 1.95 a	4.80 ± 2.54 a	5.27 ± 2.84 a	0.267	1-''none'' to 9-''very high''
**Adhesion to the spoon**	**3.47** ± **1.67 b**	**3.07** ± **1.48 b**	**4.60** ± **2.44 ab**	**6.53** ± **2.73 a**	**5.00** ± 2.**50 ab**	**0.001**^a^	1-''none'' to 9-''very high''
**Surface** **Smoothness**	**8.07** ± 1**.12 a**	**7.53** ± **2.16 ab**	**6.93** ± **2.24 ab**	**5.60** ± 2**.89 b**	**5.47** ± 2**.53 b**	**0.009**^a^	1-''none'' to 9-''very high''
**Artificial smell**	**5.33** ± **2.33 ab**	**3.8**0 ± 2**.17 b**	**4.60** ± 2.**44 ab**	**6.**53 ± 2**.00 a**	**6.47** ± 2.0**0 a**	**0,005**^a^	1-''none'' to 9-''very high''
**Natural smell**	**4.53** ± **2.94 ab**	**5.40** ± **2.09 ab**	**5.60** ± **2.24 a**	**3.13** ± 1**.96 b**	**3.33** ± **2.09 ab**	**0.012**^a^	1-''none'' to 9-''very high''
**Fruity smell**	**4.80** ± **2.17 ab**	**5.80** ± **2.29 a**	**6.20** ± 2**.17 a**	**4.93** ± 2**.43 ab**	**3.40** ± **2.12 b**	**0.016**^a^	1-''none'' to 9-''very high''
Smooth texture	8.27 ± 1.00 a	8.40 ± 1.08 a	7.80 ± 1.42 a	7.53 ± 1.20 a	7.87 ± 1.59 a	0.374	1-''none'' to 9-''very high''
Melting	7.80 ± 0.98 a	8.20 ± 1.11 a	7.87 ± 1.15 a	7.40 ± 1.40 a	6.80 ± 1.90 a	0.077	1-''none'' to 9-''very high''
**Sweet taste**	**6.87** ± 1**.09 a**	**6.73** ± **2.08 a**	**7.33** ± 1**.35 a**	**5.0**0 ± **2.97 b**	**8.07** ± **1.06 a**	**0.001**^a^	1-''none'' to 9-''very high''
Bitter taste	1.53 ± 1.09 a	1.47 ± 1.09 a	1.93 ± 2.08 a	3.07 ± 2.14 a	1.73 ± 1.53 a	0.079	1-''none'' to 9-''very high''
**Sour taste**	**3.8**0 ± **1.09 b**	**3.73** ± **1.95 b**	**2.67** ± **2.12 b**	**6.27** ± **2.72 a**	**2.47** ± 1**.41 b**	**<0.0001**^a^	1-''none'' to 9-''very high''
**Artificial taste**	**4.40** ± **2.52 b**	**4.13** ± 2**.58 b**	**3.6**7 ± **2.15 b**	**6.80** ± **2.64 a**	**5.7**3 ± 2**.67 ab**	**0.009**^a^	1-''none'' to 9-''very high''
**Overall liking taste**	**6.60** ± **1.31 a**	**6.53** ± 1.**50 a**	**6.27** ± **1.57 a**	**3.27** ± **1.95 b**	**5.93** ± **2.35 a**	**<0.0001**^a^	1-“dislike extremely” to 9-“like extremely”
**Fruit flavor**	**5.67** ± 1**.53 a**	**5.4**7 ± 1**.71 a**	**6.33** ± 1**.81 a**	**3.47** ± 2**.09 b**	**4.47** ± **2.42 ab**	**0.002**^a^	1-''none'' to 9-''very high''
**Artificial flavor**	**4.20** ± 2**.07 bc**	**4.40** ± **2.18 bc**	**3.80** ± 2**.01 c**	**7.07** ± 1**.61 a**	**6.0**7 ± 2**.79 ab**	**0.000**^a^	1-''none'' to 9-''very high''
Jelly-like texture	4.20 ± 2.56 a	3.93 ± 2.62 a	3.80 ± 2.71 a	3.60 ± 2.15 a	4.60 ± 2.50 a	0.855	1-''none'' to 9-''very high''
Adhesives Texture	3.27 ± 1.98 a	2.73 ± 1.95 a	4.00 ± 2.73 a	4.20 ± 3.08 a	5.20 ± 2.74 a	0.115	1-''none'' to 9-''very high''
Fine texture	7.47 ± 1.71 a	7.67 ± 1.45 a	6.87 ± 1.93 a	7.20 ± 2.04 a	6.53 ± 2.03 a	0.495	1-''none'' to 9-''very high''
**Granular texture**	**2.87** ± **2.96 b**	**1.00** ± 0**.00 c**	**4.67** ± 2**.30 a**	**1.0**0 ± **0.00 c**	**1.00** ± **0.00 c**	**<0.0001**^a^	1-''none'' to 9-''very high''
Oil texture	2.27 ± 2.02 a	1.73 ± 1.57 a	1.93 ± 1.48 a	2.00 ± 1.59 a	2.80 ± 2.20 a	0.569	1-''none'' to 9-''very high''
Liquid texture	5.20 ± 2.69 a	5.40 ± 2.73 a	5.27 ± 2.32 a	4.07 ± 2.26 a	3.53 ± 2.36 a	0.179	1-''none'' to 9-''very high''
Overall liking texture	6.53 ± 1.54 a	6.60 ± 2.09 a	6.13 ± 1.89 a	5.73 ± 1.88 a	6.33 ± 2.05 a	0.755	1-“dislike extremely” to 9-“like extremely”
Astringent Aftertaste	2.00 ± 2.07 a	1.67 ± 1.14 a	1.27 ± 0.77 a	2.73 ± 2.35 a	1.53 ± 1.09 a	0.152	1-''none'' to 9-''very high''
Bitter aftertaste	1.53 ± 1.09 a	1.73 ± 1.18 a	1.60 ± 1.62 a	2.80 ± 2.17 a	1.60 ± 1.14 a	0.142	1-''none'' to 9-''very high''
**Artificial** **Aftertaste**	**4.**07 ± 2**.43 b**	**3.6**0 ± **2.33 b**	**3.7**3 ± **2.21 b**	**7.1**3 ± **2.31 a**	**5.00** ± **2.71 b**	**0.001**^a^	1-''none'' to 9-''very high''
**Overall** **Acceptance**	**6.27** ± 1**.57 ab**	**7.27** ± **1.57 a**	**6.33** ± 1**.62 ab**	**2.93** ± 1.**91 c**	**5.3**3 ± **2.72 b**	**<0.0001**^a^	1-“dislike extremely” to 9-“like extremely”
Easy to swallow	8.13 ± 2.00 a	8.40 ± 1.99 a	8.00 ± 2.39 a	7.40 ± 2.47 a	7.67 ± 2.21 a	0.781	1-“completely to 9 “completely agree”.

Notes: a, b, c, d = groups within a row with different superscripts differ significantly at p ≤ 0.05; ENA – commercial energy gel with apricot flavor; RGGA – commercial energy gel with green apple flavor; ENGA – commercial energy gel with green apple flavor; ERLO – commercial energy gel with lemon-orange flavor; EEGC – commercial energy gel with cherry flavor.

a **Statistical significance**.

Table 4 shows that the most preferred samples were apple-flavored, they have a more distinct taste for the consumer, higher intensity of fruit flavor, and are more intense in sweetness as well. Rather, the presence of artificial flavors (sweeteners) was perceived negatively and, on the contrary, the unexpected graininess of the texture was perceived positively. The individual differences in the preferences achieved and their statistical difference are shown in the table (bolded p-values).

**Fig. 5:**
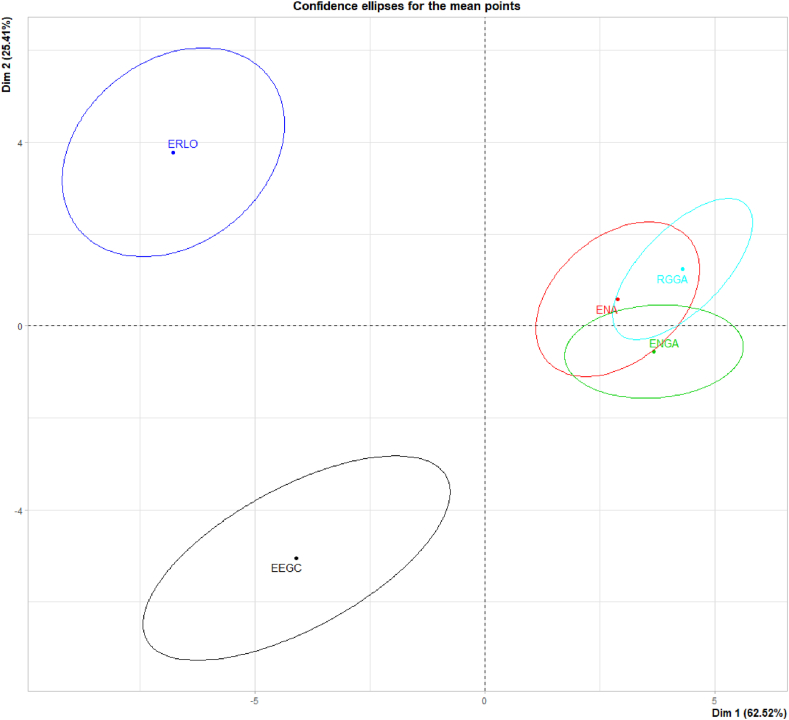
PCA analysis of complex sample profiles of the evaluated commercial gels.

PCA analysis of the treated data showed that raters perceived the ENA, ENGA, and RGGA samples as similar (preference for apple flavor, EN prefix - identical gel manufacturer). There was no statistically demonstrable difference between the samples. The size of the ellipses, on the other hand, shows a difference in the perception of the samples ERLO (lemon-orange flavor) and EEGC (cherry). The two PCs are reflected in the graph and the explained variance is 87.93 % (Fig. 5). In general, for the selected sample of the consumer panel, the acceptable group of gels is in the middle right quadrant, defined by the values and intervals from Table 3 represented by the first three samples values.

### 3.2 Temporal dominance of sensations (TDS)

Temporal Dominance of Sensations (TDS) is a sensory evaluation method used to track the dominance of sensory attributes over time during the consumption of a product. The methodology captures the dynamic changes in sensory perceptions, providing a temporal profile of dominance for each attribute. Unlike traditional static sensory tests, TDS enables the assessment of the sequence and duration of dominant sensations as experienced by the consumer. In TDS, participants are given a list of predefined sensory attributes and are instructed to select the attribute that is most dominant at each moment during consumption. This process continues until the consumption experience ends. The data collected are typically displayed in a temporal dominance curve, where the x-axis represents time and the y-axis indicates the proportion of subjects selecting each attribute as dominant. Intermittent line - the dominance rate is a key metric that quantifies the proportion of time a specific sensory attribute is perceived. To compute the dominance rate for a specific attribute at a particular time point, the number of participants who have selected that attribute as dominant is divided by the total number of participants.

**Fig. 6:**
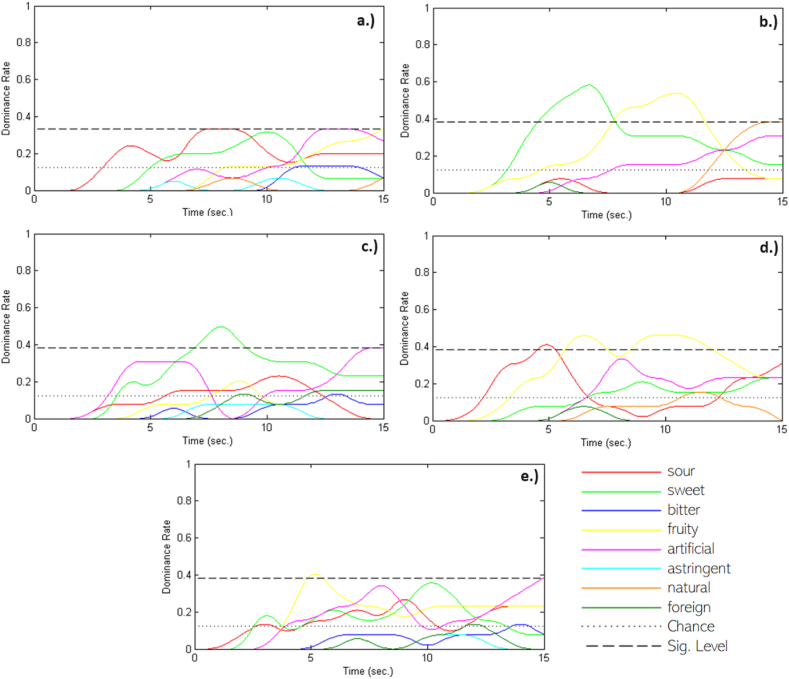
Temporal dominance sensations (TDS) curves by sample: a.) ERLO – commercial energy gel with lemon-orange flavor; b.) ENGA – commercial energy gel with green apple flavor; c.) EEGC - commercial energy gel with cherry flavor; d.) RGGA – commercial energy gel with green apple flavor; e.) ENA – commercial energy gel with apricot flavor.

A selected panel of assessors at 15-s intervals observed the dominance of the following flavors: sour, sweet, bitter, fruity, artificial, bitter, natural, and foreign. The TDS analysis results for commercial energy gels are shown in Fig. 6: ERLO (lemon-orange flavor) in Fig. 6a, ENGA (green apple flavor) in Fig. 6b, EEGC (cherry flavor) in Fig. 6c, and RGGA (green apple flavor) in Fig. 6d and RGGA (apricot flavor) in Fig. 6e. Considering the other sensory analyses, TDS was selected as a complementary informative analysis generating a more complex image of the products. In relation to the consumer analysis, it was found that people perceived a sweet taste and fruity flavor statistically significantly for apple gels (RGGA, ENGA), and a sweet taste was also perceived above the significance level for cherry gel (EEGC). The other outputs are insignificant (curves above the dominance rate). For the selected consumer panel, the use of the TDS methodology was a new type, and analysis and bias may have occurred.

### 3.3 Viscosity measurement and texture analysis

The results of the viscosity and texture analyses are shown in Table 5, and ANOVA analysis - Duncan and REGWQ tests were performed to further investigate the statistical significance, where the differences were considered significant when the p-value was greater than the significance level alpha = 0.05. Firmness represents the maximum force during the penetration testing. It is measured as the peak force recorded when the probe reaches a specified depth into the sample. The higher the values, the stronger the samples. Consistency expresses the thickness of the sample. Higher consistency means higher resistance to flow or deformation. Cohesiveness expresses the stickiness of the sample, which is manifested by the maximum negative force during probe return. A more negative value indicates stronger intermolecular forces. During testing, the work of cohesion evaluates the ability of the sample to maintain integrity and resist deformation [80].

**Table 5:** Ducan and REGWQ tests for viscosity and texture parameters.

	Dynamic viscosity (Pa·s)	Firmness (g)	Consistency (g.sec)	Cohesiveness (g)	Work of Cohesion (g.sec)
**EEGC**	1.840 a	53.790 a	919.325 a	−20.220 d	−211.975 d
**ERLO**	1.410 b	52.735 a	871.880 b	−18.265 c	−185.700 c
**ENA**	0.870 c	45.335 b	774.360 c	−15.415 b	−133.680 b
**RGGA**	0.885 c	42.745 b	743.260 d	−14.245 a	−113.845 a
**ENGA**	0.915 c	44.150 b	765.500 cd	−15.950 b	−139.170 b

Notes: a, b, c, d = groups within a column with different superscripts differ significantly at p ≤ 0.05; EEGC – commercial energy gel with cherry flavor; ERLO – commercial energy gel with lemon-orange flavor; ENA – commercial energy gel with apricot flavor; RGGA – commercial energy gel with green apple flavor; ENGA – commercial energy gel with green apple flavor.

Authors [81] developed date-based sports energy gels with the addition of xanthan gum (0.5 % w/w) as the gelling agent. They have reported that the hardness of the gels ranged from 1.11g to 1.27g depending on the dates cultivars used. The viscosity ranged from 11.417 to 8.371 Pa s (11417–8371 mPa s). The authors reported that consumers prefer softer gels, as evidenced by a lower range of hardness values. This hypothesis was also confirmed in our study (which used the same methodology), as RGGA gel was the most liked gel by the consumers and showed the lowest firmness values.

### 3.4 Food neophobia, measuring emotions by neuroimaging and face recognition

The evaluators expressed their opinions on the statements presented in Table 6, using a 7-point Likert scale ranging from strongly disagree (7) to strongly agree (1). Neophiliac items are marked with " ∗ " and the answers to these questions were reversed in the evaluation, where the individual FNS values for each respondent were calculated as the sum of the given ratings, hence the FNS scores ranged from 10 to 70. According to the method described by Olabi et al. [82], the respondents were divided into food neophilic (≤16.4), neutral (16.5–38.5), and food neophobic (≥38.6) categories.The Maximum likelihood extraction method was used in combination with the Geomin rotation in the exploratory factor analysis for the evaluation of FNS data (see Table 6). The analysis showed that the scale items form one dimension, similar to the results of the study by Paupério et al. [83] and the original version of the FNS [44].

**Table 6:** Food neophobia scale, mean values, standard deviation (SD), and the results of the exploratory factor analysis.

	Mean	SD	F1
1.I am constantly sampling new and different foods.^a^	5.0	1.8	−0.322
2. I don't trust new foods.	3.3	1.7	0.485
3.If I don't know what is in a food, I won't try it.	3.0	1.6	0.208
4. I like foods from different countries.^a^	5.4	1.4	−0.481
5. Ethnic foods look too weird to eat.	3.1	1.8	0.638
6. At dinner parties I will try a new food.^a^	4.8	1.9	−0.605
7. I am afraid to eat things I have never had before.	3.0	1.5	0.488
8. I am very particular about the foods I will eat.	2.5	1.4	0.333
9. I will eat almost anything.^a^	5.0	1.9	−0.285
10. I like to try new ethnic restaurants.^a^	5.2	1.5	−0.407
% Variance explained			0,198

7-point Likert scale ranging from strongly disagree (7) to strongly agree (1).

a Items for which the scores need to be reversed in the evaluation.

Among evaluators, 58 were classified in the neutral group. Additionally, a slightly larger number of respondents were classified in the neophilic group (10) than in the neophobic group (7). A similar classification was observed in another study [84], which addressed the adaptation and validation of the FNS for Hungary (N = 500), which represents the neighboring country of Slovakia, from where the part of respondents in our study originated.

In the current study, we used measurements based on electrical brain activity and facial expression measurements. Based on the measurement of electrical brain activity, processing of the recorded data, and after excluding outliers, a median score (engagement, arousal) for the selected emotions was obtained (Table 7). The bias score in measuring the impact of samples on emotional response (engagement, arousal). EEG has been proposed for assessing consumer preferences related to hedonic valuation in food. This technique supports traditional sensory measurements by directly capturing implicit physiological and emotional responses [[85], [86], [87]]. Despite adhering to standards and procedures [51,52] these values are only indicative and may not provide a completely accurate view of the emotions [88]. No significant differences within and between subjects’ effects were found when engagement was evaluated. The ERLO sample obtained the highest emotional engagement score (0.89), potentially influenced by respondents disliking the taste or struggling to identify the flavor [88]. These findings are confirmed by sensory testing, where the ERLO sample was rated the worst in terms of overall acceptance in the sensory evaluation (the lowest score (2.93)). The ENA sample (0.80) elicited the least emotional involvement based on the median score, which may have been due to the similarity of this sample to the previous ones. This is confirmed by PCA analysis of which data showed that the ENA, ENGA and RGGA samples were perceived similarly which corresponded with the composition stated by the manufacturer on the packaging of the gels, as these gels showed similar composition.

**Table 7:** Measurement of electrical brain activity.

Samples	Engagement	Excitement
ERLO	0.89 ± 0.05	0.42 ± 0.25
ENA	0.80 ± 0.09	0.39 ± 0.18∗∗∗
ENGA	0.86 ± 0.08	0.38 ± 0.26
EEGC	0.83 ± 0.08	0.40 ± 0.27∗
RGGA	0.83 ± 0.08	0.38 ± 0.24∗∗
*P*-values	0.966	0.001

∗*p* < 0.05, ∗∗*p* < 0.01, ∗∗∗*p* < 0.001.

On the one hand, excitement plays a crucial role as it signifies an elevated level of active engagement and anticipation when assessing the impact of samples on emotional response. When a consumer experiences arousal, various emotions are felt more intensely, significantly influencing the decision-making process and preferences [89]. Conversely, elevated arousal levels may be linked to food refusal in cases of food neophobia [90]. Based on the median, excitement showed significant differences (*p* < 0.05) within and between subjects’ effects. ERLO resulted different from EEGC, RGGA, ENA, and ENGA different from ENA. These findings are also consistent with the sensory evaluation of the samples as in particular the ERLO and EEGC samples were evaluated differently. The highest level of arousal for the ERLO (0.421) and EEGC (0.400) samples was found, while RGGA, ENGA, and ENA showed intermediate values. An interesting finding is that the highest levels of arousal were recorded for the samples that the lowest scores in terms of overall acceptance were recorded EEGC sample (5.33) and ERLO (2.93). This could result from lower recognition ability or associations evoked by the samples. Experimental and survey studies indicate that food novelty, intense flavors, perceived danger, unfamiliarity, or unusual ingredients can trigger unpleasantly high levels of arousal [91,92]. The high variance in respondents' arousal levels is noteworthy, potentially linked to the sample representation in FNS score and emotional differences, including documented cultural variations in emotional arousal [93].

In addition to electroencephalography, unconscious feedback was also sensed in the respondents by measuring microemotions based on facial expressions. Numerous researchers, including Charles Darwin (1872), have delved into the connection between facial expressions and emotions [94]. Ekman and Friesen (1971) established a connection between basic emotions and facial expressions, introducing the Facial Action Coding System (FACS) to encode facial muscle movements [95]. This method provided valuable insights into emotional valence. Recognizing cognitive biases in rating perceptions on a scale [96,97], using implicit intentional facial expressions could mitigate these biases. Facial expressions, intentionally or unintentionally used in human interactions, convey valence information rapidly [98]. Limited studies have explored the combination of facial expression analysis and autonomic nervous system responses in a food context [[99], [100], [101]].

ANOVA showed significant differences (*p* < 0.05) between EEGC and ERLO. In this case, based on the mean of the medians, respondents perceived the EEGC sample most positively (0.04) and the ERLO sample most negatively (−0.01).

The observed differences in facial expressions between edible gel samples confirm that Facereader 7 can be an effective tool for implicit feedback recognition, similar to the study [99] investigating orange juice.

The valence found according spontaneous facial expressions (especially "disgusted" and "neutral") allowed a clear distinction between “favorite” and “unpopular” samples (in terms of overall acceptance). The “unpopular” sample with lowest score (ERLO) elicited significantly more intense negative facial expressions and less neutral facial expressions than the “popular” sample with highest score (RGGA). The popular sample elicited only small changes in spontaneous facial expressions (see Table 8). This is in line with the observations made by Refs. [102,103], indicating that facial expressions serve as a reliable indicator of dislike but may not be as indicative for liking. Additionally [104], found that adult chewing muscles show greater responses to disliked than to preferred ones. Intriguingly, when facial expressions were measured implicitly, less favoured samples (EEGC, ENA) were perceived more positively compared than explicitly rated samples This phenomenon may be attributed to smiling during tasting, possibly linked to surprise or disliking the flavor. Similar effects were noted in other studies [100,101]. It may also indicate that respondents were unable to identify the samples and masked their surprise with a smile.

**Table 8:** Measurement of valence by Face reading Technology.

Samples	Valence
ERLO	−0.01∗ ± 0.032
ENA	0.02 ± 0.053
ENGA	0.00 ± 0.054
EEGC	0.04 ± 0.042
RGGA	0.03 ± 0.047
*P*-values	0.009

∗*p* < 0.05, ∗∗*p* < 0.01, ∗∗∗*p* < 0.001.

Current study compared the implicit feedback (valence) with the classification of respondents based on FNS score (neophilic, neutral and neophobic). Based on a *t*-test (p-value = 0.32), was found that there were no statistically significant differences between the emotional response classifications of the respondents based on FNS score. This finding may be linked to the relatively low representation of neophilic (13 %) and neophobic (9 %) in our sample or may confirm a result also found in another study that used EEG to detect food neophobia [38], where differences in attention allocation did not lead to varying emotional responses for all stimuli.

### 3.5 Limitations of the current study and future directions

A limitation of the study is that it is based on little qualitative research. The questionnaire used in the sensory analysis was developed based on the available literature on sensory evaluation of similar products. The motivation was to identify important sensory properties in the evaluation of gel products. However, the length of the questionnaire and the scales used may have resulted in participant questionnaire fatigue. In future research, we recommend using a questionnaire containing fewer more general attributes and the use of a suited validated scale. Neuroimaging and biometric data may have been affected by repeated assessments, the mood of the respondents, and also the weather. The evolution of consumer neuroscience in food research is likely to demand more effective integration of non-conscious metrics with cognitive and machine-generated data, such as those related to food viscosity or other technical measures. This integration is crucial for making predictive analyses regarding the factors driving various consumer responses, including purchase intent. Nevertheless, the study provides insights that are useful for other food developers who should do the same to achieve a solid foundation of information in terms of consumer preferences. We further recommend collaboration between researchers and edible gel manufacturers, as exemplified by the national APVV project. Incorporating feedback from users in the early stages when prototyping a new formulation provides important information on the shortcomings and specificities of the formulation in terms of consumer perception, which could then be refined prior to actual production and marketing.

Future research in terms of sensory research should focus on investigating the effect of viscosity of gels. Similarly, research using neuroimaging and biometric tools certainly needs improvements in terms of the tools used (multi-channel EEG, use of a GSR device to measure galvanic skin resistance instead of Facereading), the methodology (in the case of electroencephalography, focus on measuring cognitive evoked potentials in addition to power spectrum analysis of ERPs) the repetition of the research (linking sensory and neuroimaging research), and taking into account fatigue and weather.

As technology advances, becoming faster, better, and more cost-effective, the future of neuroscientific methodologies in consumer and market research will continue to unfold. However, the application of such powerful technologies also necessitates a sense of responsibility. Extensive research will be essential to validate emerging methodologies and theories, ensuring continued positive progress in the ever-expanding realm of applied consumer neuroscience. Such a challenge for food consumer science is also presented by The European consortium COMFOCUS, which seeks a higher level of harmonization of measurement and research protocols in the use of emerging methods [105].

## 4 Conclusion

The results of this study suggest that consumers perceived edible gels as convenient and easy to consume, making them a promising option for individuals, especially the elderly or those with swallowing difficulties. An important finding is that the combination of sensory, biometric, and neuroimaging methods in food research can be very useful, provided that the correct research objective is established. This approach can provide a deeper understanding of the factors influencing preferences and complement the conventional tools currently in use.

## CRediT authorship contribution statement

**Jakub Berčík:** Writing – original draft, Validation, Supervision, Software, Project administration, Methodology, Investigation, Funding acquisition, Formal analysis, Data curation, Conceptualization. **Vladimír Vietoris:** Writing – original draft, Supervision, Project administration, Investigation, Funding acquisition, Formal analysis, Conceptualization. **Melina Korčok:** Writing – original draft, Resources, Investigation, Formal analysis, Data curation. **Adriana Rusková:** Resources, Data curation. **Ján Durec:** Methodology. **Katarína Neomániová:** Writing – review & editing, Visualization, Resources.

## Informed consent statement

Informed consent was obtained from all subjects involved in the study.

## 5 Institutional review board statement

Ethical approval was obtained from the Ethics Committee at Slovak University of Agriculture in Nitra. The approval is registered under reference 2022–016.

## Data availability statement

Data available on request due to restrictions, e.g., privacy or ethics.

## Funding

This research was funded by the research grant 10.13039/501100005357Slovak Research and Development Agency under the contract No. APVV-20-0078 “Food development and its application edible gel based for the target segment of the aging population” and European Union's 10.13039/501100007601Horizon 2020 research and innovation programme under Grant Agreement No. 101005259 project COMFOCUS.

## Declaration of competing interest

The authors declare that they have no known competing financial interests or personal relationships that could have appeared to influence the work reported in this paper.
